# Aromatic Herbs as a Source of Bioactive Compounds: An Overview of Their Antioxidant Capacity, Antimicrobial Activity, and Major Applications

**DOI:** 10.3390/molecules30061304

**Published:** 2025-03-14

**Authors:** Leontina Grigore-Gurgu, Loredana Dumitrașcu, Iuliana Aprodu

**Affiliations:** Faculty of Food Science and Engineering, Dunarea de Jos University of Galati, 111 Domneasca Street, 800201 Galati, Romania; leontina.gurgu@ugal.ro (L.G.-G.); loredana.dumitrascu@ugal.ro (L.D.)

**Keywords:** aromatic herbs, bioactive phytochemicals, essential oils antioxidant activity, antimicrobial activity, applications

## Abstract

Many aromatic herbs are conventionally used for flavoring various foods, but receive wide attention because of the variety of health-related properties. The aromatic herbs can be used either fresh or as dried powders and in the form of extracts, essential oils, or purified metabolites. In this review, the main functional properties, in terms of antioxidant and antimicrobial properties, and the applications of some of the commonly used aromatic herbs from the Lamiaceae family, are discussed. Herbs like oregano, rosemary, sage, thyme, summer savory, marjoram, and basil possess high levels of bioactive phytochemicals. They are particularly rich in phenolic acids, flavones, phenolic diterpenes, and flavanones, with various beneficial effects. The phytochemical profile of aromatic plants is highly influenced by genetic factors, environmental conditions, and their interaction. In cases of the extracts and essential oils, the extraction method has a strong effect on the final composition of the herb products. Most of the applications of these aromatic herbs are related to their antioxidant, antimicrobial, and flavoring properties. In particular, aromatic herb extracts and essential oils have multiple applications in fields like food, feed, pharmaceutical, cosmetics, biopesticides, and textile industries.

## 1. Introduction

The leaves of aromatic plants, which are separated from other plant organs like roots, stalks, bulbs, barks, seeds, etc., and are used for seasoning and flavoring various foods, are typically known as herbs [1]. Spices, on the other hand, are obtained upon the drying of various parts of the aromatic plants, except for leaves [1,2]. Besides the culinary importance when used for seasoning, or as flavoring and coloring agents, many herbs and spices may help increase the shelf life of several foods and are traditionally used for various medical purposes. In particular, many herbs are rich in phytochemicals, which might ensure health-related benefits because of some important biological properties, such as antimicrobial activity, antioxidant capacity, anti-inflammatory and antitumor effects, etc. [1]. Because of the general lack of side effects at low doses, nowadays, consumers are interested in using different types of herbs-based products, which might improve the quality of life and maintain human health. However, overconsumption was reported to result in toxicological effects [1].

The present review is focused on seven commonly used leafy aromatic herbs from the Lamiaceae (Labiatae) family, namely sweet basil (*Ocimum basilicum* L.), sweet marjoram (*Origanum majorana* L.), oregano (*Origanum vulgare* L.), rosemary (*Rosemarinus officinalis* L.), sage (*Salvia officinalis* L.), thyme (*Thymus vulgaris* L.), and summer savory (*Satureja hortensis* L.). In particular, the main bioactive compounds, namely the fixed or volatile secondary metabolites found in the aerial parts of the aromatic herbs, their functional properties, with particular attention to antioxidant and antimicrobial properties, and the main applications of the fresh or dried plants, their extracts, and essential oils are presented in the paper.

Oregano, basil, and thyme are the most widely accepted aromatic plants, with the major consumers being the American and European markets. On the other hand, the popularity of rosemary, savory, sage, and marjoram varies in different parts of Europe and the USA, depending on the local food habits [3]. The aerial parts of these aromatic herbs are typically used fresh or after drying for directly seasoning different types of foods, or for preparing extracts and essential oils (Table 1).

The aromatic herbs have been consumed for centuries, and most of their applications are related to flavoring properties and enhancing food stability and safety, because of their antioxidant and antimicrobial properties, respectively. The functional properties of the aromatic herbs are highly related to their chemical composition. It should be noted that the phytochemical profile of aromatic plants is highly influenced by genetic factors, environmental conditions, and genetic x environmental effects [5].

In cases of extracts and essential oils, the procedure applied for the extraction of herb components has a strong effect on the final composition of the herb products and consequently on their properties. The extraction efficiency is dependent on several factors like cultivars, chemotypes, growing and harvesting conditions, or the extraction method [6,7]. Usually, the extraction of the bioactive phytochemicals involves the use of green solvents (water, ethanol, glycerol, isopropanol, deep eutectic solvent, supercritical carbon dioxide) or other organic solvents like acetone, hexane, chloroform, ethyl acetate, etc. [8]. The selection of the solvent and extraction method is dependent on the desired profile of components in the extract [9,10]. For example, water can be used to extract hydrophilic components, while superheating makes water a good candidate for extraction of lipophilic compounds such as essential oils, due to the reduced polarity of the water between 100 and 374 °C. Ethanol promotes the extraction of polyphenols and triterpenes, isopropanol can be used for the extraction of oils, alkaloids, gums, and natural resins, while supercritical carbon dioxide favors the extraction of lignans and essential oils [8]. Regarding the organic solvents, acetone can be used for alkaloids and oils extraction, ethyl acetate showed excellent results in the extraction of phenolic compounds, and methyl acetate is used for the extraction of phytosterols and tocopherols. Cyclohexane is frequently used for fats, waxes, and oil extraction, while benzene can be used for the extraction of volatile oils, phytosterols, alkaloids, and flavonoids [8].

**Figure 1:**
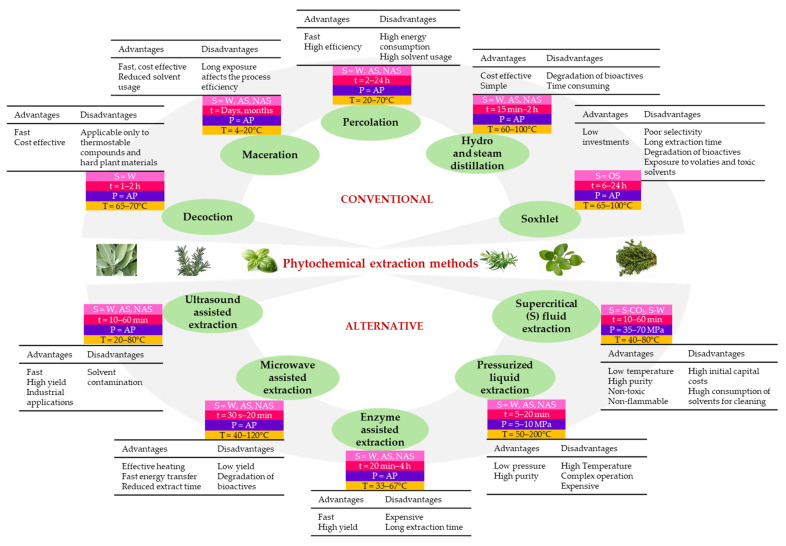
The main advantages and disadvantages of the conventional and alternative methods applied for phytochemical extraction [8,11]. S—solvent, W—water, AS—aqueous solvents, NAS—non-aqueous solvents, OS—organic solvents, t—time, P—pressure, AP—atmospheric pressure, T—temperature.

The main methods employed for the extraction of the herb’s constituents can be grouped into conventional and alternative methods, as presented in Figure 1. Most of them possess several advantages and disadvantages, the selection of an efficient extraction method being dependent both on the herb material and operational conditions (Figure 1). Conventional methods include maceration, decoction, percolation, hydro-distillation, steam distillation, and Soxhlet extraction. Maceration is applied to prepare tinctures, extracts, and concentrated infusions [8]. Percolation is frequently applied for the extraction of essential oils of the herbs, and decoction is applicable to extract saponins, tannins, and flavonoids from the hard part of the herb plants, such as the case of rosemary. Soxhlet extraction is not recommended to be used on samples with high water content, as it results in the degradation of some bioactive compounds like flavonoids [11]. Most of the conventional extraction methods are cost-effective and very simple to operate with. However, the main limitations are associated with reduced yield, long extraction time, high volumes of solvents, toxicity of some solvents, and environmental pollution [7]. These drawbacks can be minimized by using alternative methods such as ultrasound-assisted extraction, microwave-assisted extraction, enzyme-assisted extraction, pressurized fluid extraction, or supercritical carbon dioxide extraction (Figure 1). These techniques are considered eco-friendlier, and many of them offer the advantage of not altering the structure of the most bioactive molecules [7]. For instance, the extraction time of basil essential oil with solvent-free microwave extraction was reduced by 50% when compared with conventional hydro-distillation [12]. The hydro-distillation of essential oils from rosemary promoted the extraction of to a higher extent of the monoterpene hydrocarbons, while the microwave-assisted hydro-distillation favored the extraction of the oxygenated compounds [13]. Compared with the conventional methods, for most of the alternative methods, the extraction generates extracts with superior yield, while the extraction time and total costs are reduced (Figure 1). For instance, the ultrasound-assisted extraction was more efficient compared to the conventional extraction for the recovery of phenolic compounds from marjoram. Thus, the extracts obtained by ultrasounds were two-fold higher in polyphenols than those generated by conventional methods [14]. On the other hand, to prevent the degradation of bioactive compounds, the temperature during ultrasound treatment should not exceed 40 °C [8]. The supercritical fluid extraction of marjoram and sweet basil generated essential oils with anti-inflammatory properties and good potential to be used in the prevention of atherosclerosis [15]. Recent reviews of da Silva et al. [6], Shamkar et al. [7], and Kumar et al. [8] provide great details on the particularities, advantages, and disadvantages of the advanced extraction techniques of the bioactive compounds from different aromatic herbs.

## 2. Functionality of the Aromatic Herbs

The applications of fresh aromatic herbs are mostly related to their flavoring properties. On the other hand, the extracts, powders, and essential oils concentrate significant amounts of phytochemicals with important biological properties like antioxidant capacity, antimicrobial activity, anti-inflammatory properties, anticancer and antiproliferative properties, antiplatelet activity, cardioprotective activity, etc. [1,11], and are widely explored in pharmaceuticals and cosmetics, in addition to the food and feed industries.

The main types of chemical constituents and the major compounds responsible for the flavor of various aromatic herbs are listed in Table 2. The nutritional value of most aromatic herbs is mainly due to the proteins, vitamins, and mineral contents, whereas the pleasant flavor is associated with the presence of high amounts of volatile oils [3]. The protein content of most dried aromatic herbs is over 9 g/100 g d.w., with the only exception concerning the rosemary with 4.9 g/100 g d.w. [3]. The fat content ranges between 4.0 and 15.3 g/100 g d.w., with the lowest and highest contents found in basil and rosemary, respectively [3]. In addition, the fat- and water-soluble vitamins (C, A, B1, B2, B3, B5, B6, B9, E, and K) and minerals (Fe, Ca, Mg, P, Mg, Na, K, and Zn) contribute to the high nutritional value of the herbs [3,16].

The plants of the Lamiaceae family are rich in natural bioactive phytochemicals, with powerful antioxidant activity, better than that of synthetic antioxidants [3]. These phytochemicals are useful for a bunch of in vitro and in vivo applications, such as impeding the oxidative reactions occurring in fat-rich foods, and delaying the progress of many diseases, respectively [14]. The primary antioxidants found in aromatic herbs are the phenolic compounds (Table 2). A linear relationship was reported between the total phenolic content of the herbs and their antioxidant properties [3]. As indexed by the Phenol-Explorer database [21], the total phenol content (Folin assay) of the herbs varies in a rather wide range, with the lowest content registered for dried thyme (1815 mg/100 g d.w.) and the highest for basil and summer savory (4318 mg/100 g d.w. and 4512 mg/100 g d.w., respectively). The herbs of the Lamiaceae family have important amounts of phenolic acids, flavones, phenolic diterpenes, and flavanones. Among the phenolic acids, of particular interest is rosmarinic acid, a dimmer of the caffeic acid, which was reported to prevail in the aromatic herbs considered in the present study, except for marjoram (Table 2). The content of rosmarinic acid in the dried herbs varies between 308 mg/100 g d.w. in basil and 987 mg/100 g d.w in rosemary [21]. In addition, the lipophilic phytocompounds like carvacrol, thymol, carnosic acid, eugenol, linalool, etc., which are part of the essential oils, have an important contribution to the antioxidant properties of aromatic herbs. The natural antioxidants can contract the reactive oxygen species, owing to their redox properties and capacity to interfere with the production of these harmful compounds. The mechanisms involved include direct scavenging of the free radicals, chelation of the transition metals, and quenching of the singlet oxygen [34]. By neutralizing the free radicals generated in vivo, the antioxidant compounds allow for avoiding or diminishing the damage of important biological molecules like proteins, DNA, lipids, and other small molecules. The bioactive compounds extracted from herbs of the Lamiaceae family also possess antimicrobial properties against a large spectrum of microorganisms. Their efficiency depends on different factors, the most important being the geographical area, plant species, or harvesting time (the season).

### 2.1. Basil (Ocimum basilicum *L*.)

Basil is a holistic herb, widely distributed in the regions with tropical, subtropical as well as temperate climates from Europe, and North and South America. Basil is rich in bioactive phytochemicals with documented pharmacological and therapeutic properties [16]. In addition to the high nutritional value given by fats, proteins, fat- and water-soluble vitamins and minerals (Table 2), basil is also rich in many fixed or volatile secondary metabolites, including phenols, flavonoids, anthocyanins, tannins, essential oils, and steroids [16]. Ultimately, important differences in terms of chemical composition and antioxidant properties were reported among the main green and purple basil varieties, as well as between leaves and flowers. The aerial parts of basil are good sources of essential oils, accommodating up to 200 chemical compounds, including categories of compounds like monoterpene, sesquiterpene, triterpene, oxygenated flavonoids, and other aromatic compounds [35].

Basil has very strong antioxidant activity, which was assigned to various compounds separated in the alcoholic extracts or found in the essential oils (Table 2). The main compounds with antioxidant activity found in basil are phenolic acids like rosmarinic acid, flavonoids, especially anthocyanins, as well as terpenes and terpenoids like eugenol, linalool, estragole, α-cadinol, methyl cinnamate, and α-bergamotene [17,18,19,20]. The antioxidant activity of the essential oils is highly influenced by their composition. Considering the compounds that prevail in the composition of the essential oils obtained through distillation, Dhama et al. [16] described four major chemotypes: rich in methyl-chavicol, methyl-eugenol, linalool, or methyl-cinnamate. The antioxidant compounds from basil offer efficient protection against oxidative stress, acting by scavenging free radicals and increasing the activity of the antioxidative defense enzymes such as superoxide dismutase, catalase, glutathione-reductase, and glutathione-peroxidase [16].

The antimicrobial properties of essential oils extracted from the aerial parts of basil, harvested across all four seasons, were examined by Hussain et al. [36]. The most sensitive strains to the biologically active compounds from the essential oils of winter basil were *Staphylococcus aureus* ATCC 25923 (MIC of 0.9 mg/mL) and *Bacillus subtilis* ATCC 10707 (MIC of 0.8 mg/mL) [36]. It was observed that in the two mentioned seasons, the linalool and other oxygenated compounds (e.g., monoterpenes, sesquiterpene) were present in the highest concentrations, and were considered the main compounds with activity against the tested Gram-positive bacteria [36]. Linalool is monoterpene alcohol used as a food additive, but also in pharmaceuticals and cosmetics, acting on a large spectrum of pathogenic microorganisms, such as *S. aureus* NCTC 10788, *Pseudomonas aeruginosa* NCTC 12924, *Pseudomonas fluorescens* ATCC13525, *Escherichia coli* NCTC 12923 [37,38], or *Listeria monocytogenes* ATCC 19115 [39]. The inhibitory mechanism of linalool against *L. monocytogenes* was investigated through metabolomics studies, showing essential changes in the pathways related to amino acids, carbohydrates, lipids, and nucleic acid metabolism [39].

The ethanolic extract of basil, harvested from the Dalmatian region of Croatia, showed strong activity against *S. aureus* ATCC 25923, and good activity against ermC+ *Staphylococcus haemolyticus* 231 and *Yersinia enterocolitica* 4/O:3 [40]. In the experiment conducted by Ilić et al. [41], the antimicrobial activity of *Ocimum basilicum* L. cv. ‘Genovese’, cultivated in an open field in southern Serbia, under controlled shading conditions and harvested at the beginning of both August and September, was tested. The results emphasized that the essential oil had a strong inhibitory activity against *Bacillus cereus*, *Klebsiella pneumoniae*, and *Candida albicans*, regardless of the color of nets or harvesting time [41]. Furthermore, the antimicrobial activity of the sweet basil oil against *S. aureus*, *E. coli*, and *Proteus vulgaris* was higher than that of some specific antibiotics when the blue net was used, which has a promising perspective in different domains, especially in medicine [40].

Yaldiz et al. [42] tested the antimicrobial activity of twenty basil genotypes, collected from different countries (Türkiye, Iran, the USA, Afghanistan, Macedonia, Ethiopia, Georgia, Hungary), against *B. cereus* ATCC 10876, *Micrococcus luteus* ATCC 10240, *S. aureus* ATCC 25923, *Staphylococcus epidermidis* ATCC 12228, *E. coli* ATCC 25922, *Proteus mirabilis* ATCC 25933, *P. aeruginosa* ATCC 27853, *Acinetobacter baumannii* ATCC 19606, and *C. albicans* ATCC 10231. Based on the specific antimicrobial mechanisms, the authors emphasized different practical pharmaceutical applications of some genotypes, for instance, to create a cutaneous cream ingredient due to the inhibitory action of a few genotypes against *S. epidermidis*, or to use the Dino (Türkiye) or the PI 253157 (Iran) genotypes, respectively, as important tea ingredients for the kidney health, considering their activity against *E. coli*, the most frequent causative agent of the urinary tract infections [42].

The oil extract of basil leaves harvested from Ethiopian farmland and local markets proved a much lower antimicrobial activity compared to chloramphenicol against *E. coli*, *S. aureus*, and *Aspergillus niger*. The gas chromatography–mass spectrometry (GC-MS) analysis of the crude extract showed a wide range of compounds in the essential oils, the monoterpene estragole and 1-isopropyl-4-methylenecyclohex-1-ene being the most abundant [43]. The antifungal activity of estragole against *A. flavus* spore germination and its effect on aflatoxin biosynthesis were revealed by RNA-seq analysis at a 0.125 μL/mL concentration [44]. Functional enrichment analysis of genes encoding the antioxidant- or oxidoreductase-related enzymes (such as *ctl-2*, *cta1*, *pod*, *STU21*, *2ODD21*, *hxnY*, *yanF*, *yhdF*), enzymes involved in energy metabolism pathways (such as acoC, kgd1, PDA1, hxkA, gld1) or those from the secondary metabolism were all down-regulated. In addition, the genes encoding the synthesis of the compounds from cell walls and membranes, as well as those encoding the spore germination, were also inhibited by estragole, proving the suppression of fungus growth and aflatoxin synthesis, respectively [44,45]. Further, eugenol was reported to be active against *Chronobacter sakazakii*, with maximum tolerable concentration (MTC) of 0.05% and MIC of 0.2% [46]. The main antimicrobial mechanism of eugenol involves lipid membrane fraction disturbance and further disruption, leading to the loss of intracellular proteins and other cellular components and, ultimately, cell death [47].

### 2.2. Marjoram (Origanum majorana *L*.)

Marjoram is a culinary herb characterized by a strong spicy and camphoraceous pleasant odor. It is indigenous to Mediterranean countries and is currently grown mostly in Central and Western Europe, South and North America, North Africa, and Western Asia [22]. The economic value of the marjoram mainly depends on the composition of the essential oils. The bioactive phytochemicals identified in the marjoram essential oils are monoterpene hydrocarbons, oxygenated monoterpenes, and phenolic compounds [10]. Arranz et al. [15] employed the supercritical carbon dioxide extraction and obtained essential oil with variable amounts of cis-sabinene hydrate, terpinene-4-acetate, 1-terpinen-4-ol, α-terpineol, trans-sabinene hydrate, spathulenol, caryophyllene, linalool, carvacrol, and α-bergamatone. The phenolic compounds identified in the essential oils are hydroxycinnamic acids like rosmarinic acid, ferulic acid, caffeic acid, coumarinic acid, and hydroxybenzoic acids like vanillic acid, syringic acid, and p- and m-hydroxybenzoic acid [10]. The hydroalcoholic extracts, on the other hand, accommodate high amounts of phenolic acids, flavonoids, and flavonoid glycosides, which are major contributors to the antioxidant activity of the marjoram extracts. The hydroxycinnamic and hydroxybenzoic acids play an important role in this respect. Phenolic glycosides and derivatives like arbutin, methyl arbutin, vitexin, and orientinthymonin, as well as free or glycosylated flavonoids like catechin, luteolin, quercetin, quercetin-3-O-glucoside, kaempferol, kaempferol-3-O-glucoside, naringenin, narigenin-O-hexoside, apigenin, etc., were also identified in the marjoram [10]. Thus, major contributors to the antioxidant activity of the methanolic extracts obtained from marjoram are rosmarinic acid, carnosic acid, luteolin-7-O-glucoside, carnosol, apigenin-7-O-glucoside, and caffeic acid [14].

The main widely explored biological property of marjoram (*Origanum majorana* L.) is its antimicrobial action proved against a large spectrum of bacteria and fungi. Ghazal et al. [48] tested the antimicrobial effects of two solvent extracts (methanol and n-hexane) and the essential oil obtained from dried leaves against the following strains: *Staphylococcus* spp., *B. subtilis* ATCC 6633, *Enterococcus faecalis* 29212, *E. coli*, *K. pneumoniae* ATCC 700603, *P. aeruginosa* ATCC 27853, and *C. albicans* ATCC 10231. The undiluted essential oil of marjoram (0.05 mg/mL) showed remarkable antimicrobial effects compared to the solvent extracts due to the inhibitory effect on the bacterial efflux pump. The main susceptible strains were *S. aureus* ATCC 25923, *S. aureus* MRSA ATCC 43300, and *E. coli* ATCC 25922, and all of these strains had the MIC of 0.125% [48] (Table 3). Regarding antibiofilm activity, Ghazal et al. [48] showed that sabinene and sabinene hydrate appeared to be the most effective compounds against *E. coli* ATCC 25922 and *S. aureus* MRSA, demonstrating significant activity regardless of the bacteria’s cell wall structure.

Different studies emphasized the antibacterial potential of terpinen-4-ol (TP4O), the main compound found in substantial amounts in marjoram, against food-borne pathogens or those causing various human diseases [66,67,68,69]. In vitro studies on the TP4O interaction with the cell membrane proved both bacteriostatic and bactericidal effects, regardless of the Gram classification. The MICs of TP4O against *S. aureus* MRSA and *P. aeruginosa* were calculated at 1.25% and 2.5% (*v*/*v*), respectively [69], and at 0.25% (*v/v*) for other different strains of *S. aureus* [70]. According to the study of Paudel et al. [49], the essential oils from *O. majorana* (originating from Nepal) exerted effective antimicrobial activity against a range of microbial strains, assigned to the presence of key compounds, such as TP4O, linalool, and either γ-terpinene or *p*-cymene, in some samples. The sample in which *p*-cymene was present exhibited moderate antibacterial activity against *S. aureus*, their effects being less potent compared to the positive control, gentamicin. However, the antifungal properties of the same sample were more pronounced, demonstrating good activity against *A. niger* and *C. albicans* (MIC of 0.078 mg/mL), and moderate activity against *Trichophyton mentagrophytes* and *Aspergillus fumigatus* (MIC of 0.157 mg/mL) [49] (Table 3). To elucidate the action mechanism of TP4O against *S. aureus* MRSA, in silico studies were performed on the enzyme PBP2a, the penicillin-binding protein 2a, that creates resistance to beta-lactam drugs, allowing the cell wall biosynthesis due to its transpeptidase activity [70,71]. The results of the molecular docking analysis showed that the hydroxyl of TP4O interacts with the amino acid residues Ser^403^, Lys^406^, and Thr^600^, all being important for the correct anchoring of the TPO4 molecule in the active site of the PBP2a. Accordingly, the active site of the PBP2a is blocked, and the *S. aureus* is prone to become more sensitive to the beta-lactams treatments [70].

### 2.3. Oregano (Origanum vulgare *L*.)

Oregano is a perennial herb that is widely distributed, especially in the Mediterranean area. The aroma profile of oregano is characterized by a combination of spicy phenolic and minty notes [29]. The chemical composition of oregano varies between genotypes. In particular, the amount of various phytoconstituents of the essential oil was reported to differ significantly with the cultivation region, variety, harvesting period, and environmental conditions [29]. Among the compounds isolated from oregano, those with the highest commercial importance are the volatile oils, consisting mostly of terpenoids [24]. As reported by Milenković et al. [29], the essential oil of oregano consists of the sesquiterpene (24.3–31.4%), monoterpenoids (12.2–21.1%), monoterpene hydrocarbons (3.7–11.8%), and aromatic compounds (4.0–4.2%). The effective antioxidant activity of oregano was mainly attributed to the lipophilic compounds that are part of the essential oil. Alvarez et al. [23] assigned the high antioxidant activity of the essential oil obtained from oregano to the presence of phenolic monoterpenes like thymol and carvacrol (Table 2). Moreover, their biogenetic precursors γ-terpinene and p-cymene are listed among the important phytochemicals in the essential oils. Different chemotypes can accumulate up to 95% carvacrol, whereas other major components of the oregano essential oil, like thymol, γ-terpinene, and p-cymene, can be up to 60%, 24%, and 9%, respectively [29]. To be sure, hydroxycinnamic acids like rosmarinic acid, caffeic acid and its ester 5-caffeoylquinic acid, p-coumaric acid, and ferulic acid, as well as the hydroxybenzoic acids like gallic acid, syringic acid, and vanillic acid, are particularly important for the antioxidant activity of the oregano leaves and hydroalcoholic extracts [21,24,25,27]. The essential oil extracted from the Peruvian *Origanum vulgare* L. variety (Chachapoyas—2300 m above sea level) was evaluated by Tejada-Muñoz et al. [50], in terms of chemical profile and antibacterial properties, with particular attention to its effects on *S. aureus* and *E. coli*, under controlled laboratory conditions. The GC-MS chemical composition of the oregano essential oil revealed the presence of 26 compounds, primarily monoterpenes, the most abundant being 2-menthen-1-ol (36.33%), followed by linalyl acetate (9.26%), terpinen-4-ol (9.01%), and other monoterpenes, including linalool and thymol [50].

The Kirby–Bauer disk diffusion method showed that the oregano essential oil exhibited significant inhibitory activity against both *S. aureus* and *E. coli* ATCC 25922, with the largest inhibition zones observed at the highest tested concentration of 957 mg/mL [50]. Additionally, when the MBC was calculated, stronger bactericidal effects were highlighted against *E. coli* (MBC of 0.99 mg/mL) compared to both *S. aureus* isolate (3.9 mg/mL) and *S. aureus* ATCC 25923 (MBC—7.9 mg/mL) [50] (Table 3). In the study from D’Amato et al. [51], the susceptibility of different *L. monocytogenes* strains isolated from food and clinical environments to bioactive compounds from *Origanum vulgare* L. var. *hirtum* were evaluated. Notably, clinical isolates demonstrated a generally lower level of resistance to the oregano essential oils action compared to food strains, aspects confirmed by the MIC values ranging from 0.625 to 10.0 µL/mL for the food strains, higher compared to 0.312–2.5 µL/mL for the clinical strains [51] (Table 3). The same authors explained that the diminished sensitivity of *Listeria* spp. food strains to essential oils is likely due to the common inclusion of aromatic plants and spices in food processing, and this repeated exposure may lead to an adaptive increased bacterial resistance to the main active constituents from *Origanum vulgare* L. var. *hirtum* oils, represented by thymol (44.17%), γ-terpinene (26.09%), and p-cymene (16.03%) [51]. Despite this potential of bacterial adaptation, the broad-spectrum antimicrobial properties of essential oils remain valuable, particularly because they do not promote the development of resistance in the same way as traditional antibiotics. Further, the commercial essential oil from *Origanum vulgare* L. (OEO) demonstrated promising antimicrobial properties, including activity against *S. aureus* MRSA.

Cui et al. [72] evaluated the effects of OEO (0.4 mg/mL of MIC) on *S. aureus* MRSA, focusing on the impact on the bacterial cell membrane, intracellular enzyme activity, respiratory metabolism, and energy production pathways. Oregano essential oil, particularly its major component carvacrol (64.86% of the total composition), highlighted a multifaceted antibacterial mechanism against *S. aureus* MRSA, primarily through membrane disruption (proved by the leakage of small molecules, including Na^+^ and K^+^ ions, and losing intracellular components), inhibition of respiratory metabolism (proved by an increasing citric acid content due to the inhibition of the TCA cycle), and energy depletion (emphasized by decreasing the ATP content by 44.32%) [72]. In general, antimicrobial mechanisms exerted by carvacrol against bacteria cells involve several key processes responsible for bacterial cell disruption and death, including cytoplasmic membrane alterations, cytoplasmic coagulation and leakage of essential components, metabolic imbalance, and influences on the nucleic acids’ synthesis. In particular, carvacrol, along with other phenolic compounds, which have a hydrophobic nature can interact with the acyl chains of the phospholipid bilayer and can destabilize the membrane by increasing its permeability. These processes lead to the reduction in the intracellular pH and ATP depletion, as well as ATP leakage through the damaged membrane [73].

Gwiazdowska et al. [52] demonstrated that *Origanum vulgare* L. supercritical carbon dioxide (SC-CO_2_) extracts exhibited higher antagonistic activity against both Gram-positive and Gram-negative bacteria compared to their fungistatic effects, with MIC values ranging from 0.25 to 5.0 mg/mL. Interestingly, the GC-MS analysis of the essential oils from different *Origanum vulgare* L. cultivars highlighted chemotype-dependent bioactivity, which was influenced by the highest concentrations of the main compounds, such as carvacrol (’Hirtum’, ’Margarita’, ’Hot & Spicy’) or trans-sabinene hydrate (’Variegata’, ’Aureum’) [74]. The essential oils from the cultivars having the highest concentration of carvacrol exhibited similar MIC values against *Haemophilus influenzae* (0.15 mg/mL), *H. parainfluenzae* (0.15 mg/mL), *P. aeruginosa* (0.30 mg/mL), and *S. aureus* MRSA (0.6 mg/mL) [74].

### 2.4. Rosemary (Rosmarinus officinalis *L*.)

Rosemary is an evergreen camphor-scented perennial shrub, originating in the Mediterranean region, from where it spread, reaching other warm regions of the world, such as South America or the West Indies [26]. Rosemary has excellent antioxidant properties, the bioactive phytochemicals acting as metal chelators, scavengers of superoxide radicals, and lipid antioxidants [28]. The main phytochemicals from rosemary are phenolic compounds, namely phenolic acids, flavonoids, di- and triterpenes, and essential oils. The phenolic acids commonly found in rosemary are rosmarinic, caffeic, chlorogenic, and p-coumaric acids [21,25,26,27]. The flavonoids are present in very low amounts and include mainly naringin, cirsimaritin, hispidulin, apigenin, luteolin, diosmin (diosmetin 7-O-rutinoside), and genkwanin [21,26]. Among diterpenes, carnosic acid, carnosol, and rosmanol were mainly listed as strong antioxidants (Table 2). The antioxidant activity was noticed even after oxidation of carnosic acid and carnosol into o-quinones. The triterpenes of rosemary, on the other hand, are known for the anti-inflammatory and tumor-inhibitory properties.

A lower contribution of the lipophilic compounds from rosemary to the antioxidant activity was suggested by Alvarez et al. [23]. Rosemary essential oil contains various amounts of 1,8-cineole, α-pinene, camphor, borneol, camphene, p-cymene, β-pinene, and limonene, with the composition being highly influenced by environmental factors, the vegetative stage of the aromatic herb, and the extraction procedure. Depending on the predominant monoterpenoid, Aziz et al. [26] mentioned three different chemotypes that are rich in 1,8-cineole, α-pinene, or camphor. Among the lipophilic phytochemicals of the essential oil from rosemary, Alvarez et al. [23] highlighted the importance of the α-pinene (9.0–26%) contribution to the overall antioxidant activity. Ultimately, the total phenolics and antioxidant activity of the essential oil from rosemary are significantly lower compared to oregano [23].

In the study from Lorenzo-Leal et al. [54], the commercial essential oil of rosemary exhibited antimicrobial activity, effectively inhibiting the growth of *A. baumannii BAA-747* (MIC of 0.5 mg/mL), but showed no inhibition against *E. coli* ATCC 25922, *P. aeruginosa* ATCC 14210, or *S. aureus* MRSA. It seems that the lack of activity can be explained based on differences in chemotype oils at concentrations lower than 2 mg/mL. Additionally, the rosemary essential oils showed antifungal activity against *C. albicans*, with a MIC of 0.6 mg/mL [54] (Table 3). The authors also highlighted the importance of essential oil compounds, particularly terpenes and terpenoid compounds, which are known to exert antibacterial effects [54]. The GC-MS analysis of SC-CO_2_ rosemary extract indicated the methyl linoleate (66.78%) as a major compound, whereas in the essential oils’ composition, the α-pinene, camphor, verbenone, 1,8-cineole, and borneol prevailed [53]. Although there were notable differences in chemical composition, the antibacterial activity of both samples against *E. coli* and *S. enteritidis* strains was similar, with a MIC value of 2.560 mg/mL for the tested Gram-negative bacteria [53] (Table 3). In general, these compounds interact with the proteins from the cell membrane, affecting its functionality and integrity, or can alter the genetic material affecting different processes from the cell, such as electron transport, fatty acid biosynthesis, phospholipase, and esterase inhibition, etc. [75,76,77]. Four different extraction methods (maceration, infusion, Soxhlet, and ultrasound) were applied to the rosemary leaf and their impact on the antioxidant and antimicrobial properties was assessed by Macedo et al. [55]. All extracts exhibited some degree of antimicrobial activity, with the infusion and ultrasound extracts being the most effective (Table 3). Specifically, the rosemary infusion extracts inhibited the *Enterobacter agglomerans* isolated from urine samples and *E. coli* from the tracheal secretions, while ultrasound extracts showed activity against *P. aeruginosa* (MIC ≥ 6 mg/mL) and *E. coli* (MIC ≥ 25 mg/mL), also isolated from urine [55].

### 2.5. Sage (Salvia officinalis *L*.)

Sage, also known as the queen of herbs, is an evergreen perennial woody shrub, with a bitter astringent taste and strong aroma [5]. The sage leaves are widely used for preparing natural extracts and essential oils with a bunch of applications in food, cosmetics, and pharmaceutical industries owing to their antioxidant and antimicrobial activities [5,78]. Among the bioactive compounds originating from sage, the antioxidant activity was assigned to two major groups of secondary metabolites, namely the volatile terpenoids and polyphenolic compounds [5,78]. In particular, Yanishlieva-Maslarova and Heinonen [28] described the important role of rosmarinic acid, carnosol, carnosic acid, rosmanol, and rosmadial to the excellent antioxidant properties of sage. The essential oils accumulation in sage and the overall quality was reported to increase with the drought stress severity [78]. The compounds found in the highest concentration in the essential oils belong to the monoterpene class. Among the 28 different monoterpenes identified by Vasoughi et al. [78] when studying the effect of elicitors and irrigation frequency on the phytochemical traits of sage, the following were reported to prevail in the mixture: α-thujone, camphor, 1,8–cineole, β-thujone, comphene, α- and β-pinene, and borneol. The essential oils also contain smaller amounts of aroma compounds like limonene, linalool, and bornyl acetate, as well as sesquiterpenes like α-humulene, viridiflorol, and phenolic compounds [5,78]. Martins et al. [5] compared different extraction methods, namely in water through infusion and decoction and in the aqueous methanolic solution, and identified the phenolic compounds extracted from sage. They reported that the methanolic solution ensured better solubilization of phenolic compounds with stronger antioxidant activity. In the case of the aqueous extract, the decoction appeared to be more efficient compared to infusion in extracting the bioactive compounds with antioxidant activity. It should be noted that decoction ensured the recuperation of the highest concentration of phenolic compounds, whereas the infusion in water was the least effective. Ten phenolic acids and derivatives (rosmarinic, caffeic acids, rosmarinic acid hexoside, and caffeic acid hexoside) and twelve flavonoids (mainly luteolin derivatives like luteolin 7-O-glucuronide, luteolin 7-O-glucoside, luteolin diglucuronide, luteolin acetylglucoside, and luteolin 7-O-rutinoside, but also apigenin-7-O-glucoside and ispidulin glucuronide) were quantified in the extracts. The rosmarinic acid and luteolin 7-O-glucuronide were the phenolic acid and flavonoid, respectively, found to prevail in all extracts [5]. The higher antioxidant activity of the methanolic extract was assigned to the presence of caffeic acid, luteolin-7-O-glucoside, apigenin acetylglucoside, and hispidulin in higher amounts. Previous studies of Kivilompolo & Hyötyläinen [25] and Kivilompolo et al. [27], who compared different chromatography-based methods to quantify the antioxidant phenolic acids in herbs from the Lamiaceae family, showed that rosmarinic acid is the major phenolic acid in sage, followed by caffeic and chlorogenic acids. In addition, the GC-MS analysis indicated the presence of vanillic acid, gallic acid, ferulic acid, p-coumaric acid, and syringic acid in the sage.

The antimicrobial compounds from sage essential oils can act and inhibit the growth of different pathogenic microorganisms like methicillin-resistant and methicillin-sensitive *S. aureus*, *E. coli*, *P. mirabilis*, *B. subtilis*, *P. aeruginosa*, *Streptococcus pyogenes*, or *C. albicans*, with some of them being responsible for different nosocomial infections [56,57,58,59,60] (Table 3). The MIC of salvia essential oils against *S. aureus* ranged from 0.125 mg/mL to 2.853 mg/mL, while, against *Pseudomonas* spp. strains, ranged from 0.125 mg/mL to 0.29 mg/mL [56,57,58,59,60]. In a recent study, Santos et al. [61] determined the bactericidal concentrations of hydroethanolic sage extract against clinical isolates of bacteria found in post-treatment periodontitis (*E. faecalis* strains) or in the root canal of patients with apical periodontitis (*Enterococcus faecium)*. A minimum concentration of 1 mg/mL of hydroethanolic sage extract had a bactericidal effect against the *E. faecalis* clinical strain 2, while a concentration of 2.1 mg/mL was effective against the *E. faecium* clinical strain 1 [61] (Table 3). Nanotechnology is a rapidly developing area of science that enables the synthesis of nanoparticles/nanomaterials for incorporating plant extracts, offering excellent biological properties. Ciobanu et al. [79] highlighted that the sage-coated zinc nanoparticles in the dextran matrix (7ZnHAp-SD) were active only against the *E. coli* ATCC 25922 strain, with no other inhibition observed against *S. aureus* ATCC 25923, *E. faecalis* ATCC 29212, or *P. aeruginosa* ATCC 27853 at the tested concentration of 5 mg/mL.

Although the whole antimicrobial mechanism exerted by the bioactive compounds from sage against different bacteria is not well understood, Speranza et al. [80] mentioned that the cell membrane damage, which resulted in leakage of essential intracellular molecules, such as ATP and DNA, was mainly responsible for the 99.99% reduction in the pathogen population.

### 2.6. Thyme (Thymus vulgaris *L*.)

Thyme is a culinary herb that grows well in the Eurasian and northwest African Mediterranean regions [30] and has a typical spicy aroma and thymol-characteristic odor [29]. The chemical composition of the thyme is highly influenced by a variety of factors such as climate, cultivation conditions, and harvesting period. There are mainly two classes of secondary metabolites of high importance for the functionality of thyme, namely the volatile essential oil and non-volatile polyphenols [30]. The antioxidant properties of the volatile oils from thyme are excellent, comparable to α-tocopherol or butylhydroxytoluene, and significantly higher to oregano and marjoram (over 10 and 20 times higher, respectively) [29]. As reported by Milenković et al. [29], the essential oil of thyme consists of aromatic compounds, oxygenated mono- and sesquiterpenes, and sesquiterpene hydrocarbons. The essential oil accommodates high amounts of monoterpenes with powerful antioxidant activity, like thymol (8.1–9.4%), γ-terpinene (3.5–4.0%), p-cymene (2.8–3.6%), carvacrol and linalool (1–5%), and the sesquiterpene caryophyllene oxide (1.5–2.2%) [29,30]. The tannins represented by rosmarinic acid, and the free phenolic acids, e.g., caffeic acid, syringic acid, ferulic acid, and p-coumric acid are important contributors to the antioxidant activity of the thyme, in addition to the biphenyl compounds. Finally, the most important flavonoids from thyme are apigenin and luteolin, whuch are found both as aglycones or as O-glycosides and the methylated flavones [30].

Thyme essential oil has been extensively studied regarding its activity against antibiotic-resistant pathogens. Thyme essential oil has a high content of bioactive compounds, particularly thymol, carvacrol, p-cymene, and/or γ-terpinene, depending on the variety, season of harvesting, or environmental conditions. For instance, different commercial thyme essential oils (Infinite Choice—Portugal, Merck Life Science—Czech Republic) demonstrated promising activities against a wide range of reference and isolated bacteria and fungi strains, such as *S. aureus* MRSA, *Bacillus* spp., *Clostridium* spp., *Listeria* spp., *Enterococcus* spp., *Acinetobacter* spp., *Pseudomonas* spp., *Salmonella* spp., *Yersinia* spp., *Candida* spp., *Arcobacter* spp., *S. cerevisiae*, etc. [62,63] (Table 3). The lowest MIC value of 0.02% was observed for *E. flavescens*, while the highest MIC of 0.19% was reported for *B. cereus* and *E. faecalis* strains [62] (Table 3).

In previous research, Hassanzadeh et al. [81] demonstrated that thyme essential oil, at sub-inhibitory concentrations, induced a suppressive effect on *S. enteritidis* virulence genes. This effect was more pronounced for those genes involved in the invasion, helping bacteria to enter into the host epithelial cells (*hil*A) and to survive and replicate inside the macrophages (*spv)*, than for fimbrial adhesion (*sef*A). Similar observations were reported by Giovagnoni et al. [82] and Morshdy et al. [83] in the case of *S. typhimurium* treatment with thymol and carvacrol.

### 2.7. Summer Savory (Satureja hortensis *L*.)

Summer savory is an annual herbaceous shrub having a thyme-like flavor, distributed in the warm regions of both hemispheres [31,33]. The phytochemical profile of the summer savory includes secondary metabolites such as volatile oil, phenolic compounds, sterols, and tannins. As described by Boroja et al. [33], among the 20 different phenolic compounds identified in the summer savory methanol extract, the phenolic acids prevail in the aerial parts of the aromatic herb, with rosmarinic acid as the most abundant compound (25 mg/g of dried extract). Other hydroxycinnamic acids, found in concentrations at least 20 times lower with respect to the rosmarinic acid, are caffeic acid > isoferulic acid > p-coumaric acid > sinapic acid > chlorogenic acid. Another valuable biologically active compound with demonstrated antioxidant activity is syringic acid, which is a 4-hydroxybenzoic acid. Boroja et al. [33] also reported the presence of a high amount of naringenin in the summer savory extract, and negligible contents of flavones and their glycoside derivatives (apigenin and apigetrin, luteolin, and vitexin, respectively). A similar chemical profile, additionally including flavonoid aglycones (catechin and epicatechin) and glycosides (rutin, hesperidin, apigenin-7-glucoside), was reported by Chkhikvishvili et al. [84] for the ethanolic extract of summer savory. The essential oil of summer savory was also found to have good antioxidant activity, owing to the presence of dominating oxygenated monoterpenes thymol and carvacrol [32]. Other important terpenoids found in the essential oil are γ-terpinene, myrcene, p-cymene, linalool, β-caryophyllene, α-pinene, and some derivatives [31,32,33].

The biological activity of summer savory was primarily attributed to carvacrol, thymol, γ-terpinene, and p-cymene, compounds that possess strong antibacterial properties, against various bacterial strains, including *E. coli*, *S. aureus*, *P. aeruginosa*, *S. typhimurium*, *L. monocytogenes*, or *K. pneumoniae* with MIC values ranging from 0.25 mg/mL to 5.0 mg/mL [64,65] (Table 3). Fungal contamination in the food industry, particularly by *Aspergillus flavus* and *Aspergillus parasiticus*, can lead not only to food spoilage, but also to the production of aflatoxins, which are hepatocarcinogens in both animals and humans. The essential oil of *Satureja hortensis* L. demonstrated significant antifungal activity against *A. flavus*, with complete inhibition observed at 350 and 500 ppm [85]. *C. albicans* is a fungal pathogen commonly found in oral lesions of HIV-infected patients, capable of forming biofilms, which are resistant to antifungal treatments. In a study by Sharifzadeh et al. [86], the antifungal effects of summer savory essential oil on *C. albicans* planktonic and biofilm cells were investigated. Thymol, as the main compound, was found to significantly inhibit *C. albicans* biofilm formation and reduce the cells’ metabolic activity, causing morphological changes in biofilms, and disruption of its three-dimensional structure, making this oil a potential therapeutic agent [86].

## 3. Applications

**Figure 2:**
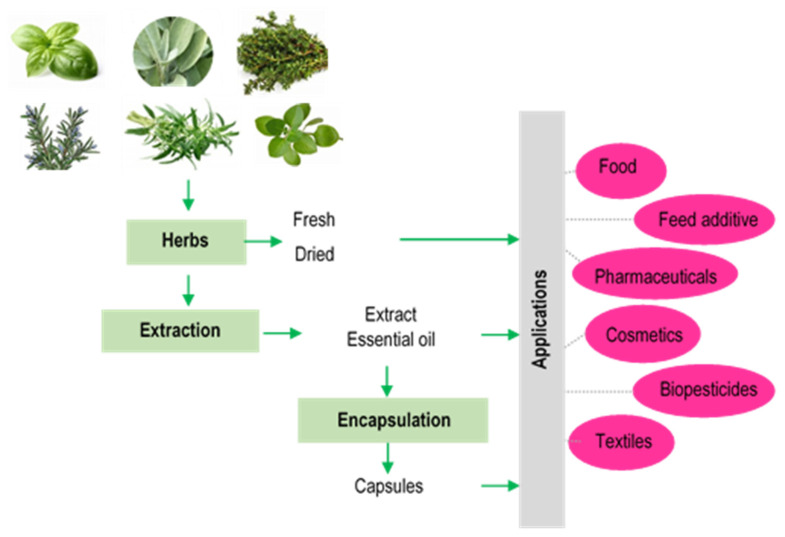
The main applications of herbs.

Herbs can be used in various applications, ranging from food to the pharmaceutical and textile industries. Depending on the applications, the bioactive compounds from herbs can be extracted from different plant parts (flower, leaves, stems, etc.) that can be added directly to different products in the form of powders, extracts, and essential oils, or after encapsulation, meant to provide protection for the bioactive compounds (Figure 2).

The use of herbs in the form of extracts/essential oils in various formulations has to be carried out in agreement with the legal framework regulated by authorities like the Food and Drug Administration (FDA) in the United States, or the European Commission in Europe. For example, according to the FDA, the use of essential oils and extracts is considered generally recognized as safe. Therefore, it is not subjected to quantitative restrictions and does not require government approval prior to their use [87]. On the other hand, at the European level, the legal framework is different. For example, rosemary extract is the only extract authorized as a food additive, coded as E393 number, and can be used in several food products with specific maximum limits.

### 3.1. Applications in Food

In the food industry herbs like basil, rosemary, sage, thyme, marjoram, summer savory, and oregano are widely used due to their antioxidant, antimicrobial, and flavoring properties. Basil can be added to meat and meat products, fish, salads, dressings, or sauces [88]. Marjoram is frequently added in seasoning sausages and salamis, but it can be used in soups, salad dressings, or sauces for stewed meats and stuffing, or as a substitute for oregano or thyme [22]. Oregano is used in meat, sausages, dressings, soups, etc., while, in the form of essential oil, found applications in alcoholic beverages, baked goods, snack foods, fats, oils, or processed vegetables [89]. Rosemary and sage can be added to several foods like meat and dairy products, fish and fish products, edible oils, condiments, or jelly candies to maintain the product quality and extend their shelf life [13,90]. Thyme can be added to a variety of food products, such as meat, milk, or fish products. Summer savory is a very popular ingredient in Mediterranean cuisine that can be used in meat products such as sausages and in beans or soups, where it adds a peppery flavor to dishes and, in some cases, can replace parsley [91,92].

The aromatic herbs can be used in food applications in several forms either fresh or dried, as extracts or essential oils (Table 4). The form is dependent on the bioactive composition, food matrix composition, and desired properties. For example, the use of oregano essential oil in semi-hard cheese was effective against spoilage microorganisms; however, a concentration of 1.25 μL/mL had a bactericidal effect on lactic acid bacteria [87]. On the other hand, the use of an emulsion containing a synergistic concentration of rosemary (2.65 µL/mL) and oregano essential oils (0.07 µL/mL) in the Minas cheese altered the growth of *E coli* O157:H7, without affecting the growth of *L. acidophilus* [93]. The aqueous extract of rosemary was found to possess a higher phenolic content than ethanolic extracts, which was further selected to be incorporated into cottage cheese [94]. Kunova et al. [89] tested the use of dried oregano, rosemary, and thyme herbs, and their corresponding essential oils, as antimicrobial agents for sheep lump cheese. The authors showed that the tested dried herbs, along with their respective 1% essential oils inhibited the growth of total viable bacteria, coliform bacteria, and microscopic filamentous fungi for up to 14 days of storage under vacuum packaging. The authors concluded that dried herbs could replace their corresponding essential oil, as cost-effective biopreservatives. The antioxidant and antimicrobial properties are enhanced when increasing the concentration of herb extracts/essential oils; however, exceeding a threshold limit could have a negative impact on some foods that use starter cultures. The method applied for treating the food product with herb extracts/essential oils was found to be significant when developing products with an extended shelf life. For example, Nabigol [95] treated the strawberry surface with an emulsion containing summer savory essential oil (1200 μL/L) by fumigation, spraying, and dipping to control post-harvest diseases. Fumigation of the fruit surface with an emulsion containing essential oil ensured efficient antifungal activity against post-harvest pathogenic fungi like *Rhizopus stolonifer*, *Penicillium digitatum*, *Aspergillus niger*, and *Botrytis cinerea*. The use of summer savory-derived hydrosols to treat the fresh-cut cucumbers and tomatoes ensured antibacterial activity against toxicogenic *E. coli* O157:H7, while the water wash was not able to eliminate these bacteria [96].

**Figure 3:**
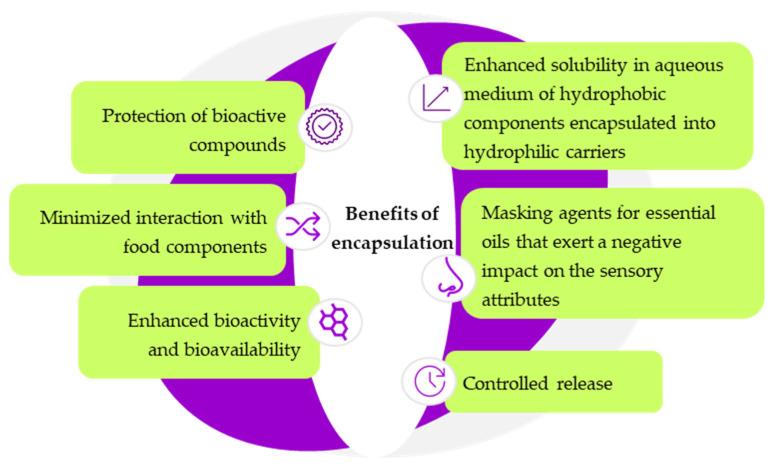
The benefits of encapsulation of essential oils (adapted from [112,113]).

One of the most common drawbacks of using herb essential oils/extracts in food applications is associated with the sensorial attributes. The concentration in which herb extracts/essential oils can be added to reach consumer acceptability is dependent on the food type and way of incorporation. For example, Maleš et al. [101] tested the possibility of using aqueous extracts from sage and thyme in juice formulations. The addition of a maximum of 10% herb extract in orange juice was well perceived by the consumers in terms of sensory attributes. On the other hand, acceptable sensorial attributes of the skimmed yogurt and Kariesh cheese were reported when marjoram and sage extract did not exceed 1% and 2%, respectively [98]. Despite their numerous food applications, most of the bioactive compounds from herbs in the form of extract or essential oils are easily prone to degradation when exposed to light, oxygen, heat, humidity, and mechanical stress, or can interact with other components of the food matrix [111]. These drawbacks are tackled by using encapsulation. Moreover, through encapsulation, the negative effects of herb extracts/essential oils on the flavor of food products are minimized, whereas the food protection efficiency is maximized, as encapsulation reduces the volatility and hydrophobicity of herb extracts/essential oils [13]. The main advantages provided through encapsulation are presented in Figure 3.

Some examples of using encapsulated extracts/essential oils in food are collected in Table 5. The encapsulation of essential oil (0.5%) from rosemary in inulin and whey protein isolate and the addition into Minas frescal cheese at the end of cheese processing increased the shelf life of the cheese by controlling the proliferation of mesophilic bacteria. On the other hand, the same concentration of encapsulated oil was not effective against coliforms [114]. The beef meat fillets containing encapsulated rosemary extract possessed increased antimicrobial and antioxidant activity, extending the shelf life of fillets to 21 days [115]. The encapsulated rosemary aqueous extract was able to provide a higher antioxidant activity during the storage of cottage cheese compared to the non-encapsulated extract [94]. Hamburgers with encapsulated thyme essential oil showed superior preservative potential than non-encapsulated samples. Thus, the use of encapsulated thyme essential oil provided significantly longer protection against thermotolerant coliforms and *E. coli* of up to 14 days, compared to the non-encapsulated oil (7 days) [116].

### 3.2. Active Food Packaging

Films and coatings have excellent barrier properties, edibility, and renewable and biodegradable characteristics, with excellent potential to replace conventional packaging materials. An edible film is associated with a thin layer formed separately and wrapped on the food surface, while an edible coating is associated with a thin layer formed directly on the food surface. In both cases, these edible packages can be either consumed with the food product or can be disposed of without affecting the environment [123]. Due to their antimicrobial and antioxidant properties, herb extracts/essential oils have been used in active packaging to enhance the shelf life and maintain the quality of both fresh and processed foods, while reducing the risks to human health and the environment [124]. The food applications range from meat and dairy products to fresh fruits and vegetables, as presented in Table 6. Despite the benefits ensured by these active packages containing herb extracts/essential oils, the films and coatings do not have the ability to possess more than just one function, which limits their use in industrial-scale production and commercialization [123].

### 3.3. Feed Additives

Herb extracts/essential oils represent an important group of phytogenic feed additives used as alternatives to antibiotic growth promotors due to their antimicrobial, antioxidant, and anti-inflammatory potential [106]. Moreover, in animal production, the bioactive compounds found in herbs, or their extracts/essential oils, can be used as sensory phytogenic products, technological additives for enhancing the quality and safety of feed, or as additives that promote animal health and welfare, by acting as immunomodulatory agents, antioxidants, digestive stimulants, and constituents that improve the performance and quality of animal products [106]. The quality of egg and meat is enhanced using herb extract/essential oils, due to their antioxidant potential that prevents lipid oxidation (Table 4). Encapsulation of bioactive components from herb extracts/essential oils prior to being used as feed additives limits the oxidative processes, enables target delivery to the intestinal tract of the animals, and masks strong odors of some herbs that limit animal acceptability [106] (Table 5).

### 3.4. Pharmaceuticals

The components found in herb extracts/essential oils present various medicinal properties that can be used to develop pharmaceuticals. Basil has antibacterial, antifungal, antiviral, cardioprotective, anticancer, hepato-renal protective activities, anti-inflammatory, and antidiabetic effects [137]. Marjoram’s pharmacological properties are associated with antioxidant, hepatoprotective, cardioprotective, anti-platelet, gastroprotective, antibacterial and antifungal, antiprotozoal, antiatherosclerosis, anti-inflammatory, antimetastatic, antitumor, antiulcer, and anticholinesterase inhibitory activities [10]. Oregano-based products can be used as stimulant, carminative, stomachic, diuretic, and diaphoretic agents. In addition to their antimicrobial effects, the antioxidant, anti-inflammatory, and anti-carcinogenic properties of thymol and carvacrol from thyme essential oils have been extensively studied [138,139]. The antioxidant properties were demonstrated by their ability to reduce oxidative stress by scavenging free radicals and reactive oxygen species (ROS), inhibiting lipid peroxidation, and activating the functions of endogenous enzymes [140]. Although the mechanisms through which the monoterpene phenols from thyme exert in vitro multi-targeted actions on various cancer cell lines are not fully understood, several studies demonstrated their ability to induce apoptosis and DNA degradation and reduce cell viability, therefore suggesting their potential as chemopreventive agents [139,141]. Sage is used due to its mild tonic, astringent, and carminative properties, while thyme has antispasmodic, carminative, emmenagogue, anthelmintic, spasmodic, laxative, stomachic, tonic, and vermifuge properties [3]. Rosemary extracts/essential oils exert anti-inflammatory capacity, neuroprotective, antidepressant, wound-healing potential, anti-obesity, and anti-skin cancer activity [26,142]. In addition, the rosemary extract may exert antiproliferative and apoptotic effects, demonstrating promising in vitro anticancer properties in various colon, breast, pancreas, and ovarian cancer cell lines, respectively [143,144,145]. Different apoptotic mechanisms of rosemary extract include the enhancement of nitric oxide production, upregulation of mitochondrial-regulated apoptosis proteins (such as cytochrome c and heat shock protein 70), as well as the upregulation of pro-apoptotic Bax, cleaved-caspase 3, and proteins associated with endoplasmic reticulum stress [144,145]. Furthermore, among its bioactive compounds, rosmarinic acid has shown low toxicity, with a lethal dose (LD50) of 561 mg/kg, while carnosic acid has an LD50 of 7100 mg/kg in acute toxicity studies in mice [146,147]. Thus, the rosemary was classified by the FDA as GRAS (generally recognized as safe) and has been widely used in traditional medicine, to alleviate a variety of mental illnesses including epilepsy, nervous agitation, depression, etc. [148].

Sage has a long-standing history in alternative medicine, being used to treat dyspepsia, stomatitis, inflammation, and pharyngitis, and to alleviate the discomfort of patients with advanced oral cancer as palliative care [149,150,151]. Moreover, sage has cognitive-enhancing and cardiovascular health effects, anti-hyperglycemia/hyperlipidemia properties, cytotoxicity/anticancer potential, and is a skin curative, as well as the potential for treating Alzheimer’s disease [152]. Summer savory is frequently used in the treatment of cramps, nausea, indigestion, and gastrointestinal disorders, with good results being also reported for treating inflammation-related diseases and prevention of oxidative-stress-related injuries [33]. In vitro treatment of cells with *S. hortensis* extracts induces the regulation of pro-inflammatory cytokines expression and upregulates the genes encoding antioxidant enzymes (superoxide dismutase, catalase) protecting the cells from oxidative damage, offering potential benefits in aging, cancer, and neurodegenerative diseases. Furthermore, studies on human cancer cell lines have shown that carvacrol influence the expression of genes involved in apoptosis and cell cycle regulation, suggesting its potential as an adjunct to traditional cancer therapies [153].

Ultimately, all herb extracts/essential oils, regardless of the form in which they are used (extracts, oils, powders, water), must pass the animal and clinical tests prior to use in pharmaceutic and cosmetic formulations.

### 3.5. Cosmetics

In the cosmetic industry, aromatic plants, such as sage, thyme, rosemary, and oregano, represent an attractive alternative for developing eco-sustainable products with dermato-cosmetic properties and natural marketing image [154]. The active ingredients from herb extracts/essential oils are widely used for developing skin and hair care products like moisturizers, lotions, skin cleansers, conditioners, antidandruff products, and lipsticks [155]. Due to their antimicrobial activity, cosmetics containing herb extracts/essential oils provide protection against microbial infections while preserving the cosmetic product [154]. In addition, herbs like sage exhibit excellent anti-aging and anti-wrinkle properties, whereas thyme, oregano, and basil possess high antioxidant activity, which provides sun protection [105,155]. Rosemary-derived ingredients are frequently used in cosmetics as skin conditioning agents or as fragrance ingredients [156] (Table 4). The flower and leaf of rosemary extract are used in cosmetics as antioxidants, whereas the leaf powder is used for its flavoring characteristics [156]. The use of encapsulated forms of herb extracts/essential oils for cosmetic purposes enables the incorporation of both hydrophilic and hydrophobic bioactive compounds, controlled release, and an increase in the phytochemicals’ stability and bioavailability [122] (Table 5). For example, Montenegro et al. [122] showed that gels containing nanoparticles loaded with rosemary essential oil enhanced skin elasticity and hydration, compared with non-encapsulated essential oil. Herbs that contain fragrance allergens, like those from rosemary, must be declared on the label if allergen concentration exceeds a threshold limit of 0.001% in leave-on (e.g., body lotions, etc.) and 0.01% in rinse-off products (shower gel, etc.) [156]. The herb extracts are used up to 10% in the body and hand products, and up to 3% in eye shadow applications [156].

### 3.6. Biopesticides

The use of herb extracts/essential oils as natural alternatives to chemical pest control increased in the last few decades due to their efficacy, multiple mechanisms of action, low toxicity, and safe use [157]. Herb extracts/essential oils exhibit a high effectiveness against several pests, with some examples provided in Table 4. Basil essential oil was found to be suitable for use as an insecticide and fumigation agent in controlling grain storage pests (grain weevil and red flour beetle) [158] and rosemary essential oil was effective in controlling the spider mites on greenhouse tomato plants [108], whereas thyme essential oil controlled the *Glyphodes pyloalis* W in mulberry orchard [109]. The encapsulation of herb extract/essential oil was shown to enhance the stability and potency of biopesticides (Table 5). The use of nanotechnology presents a high potential to develop biopesticides, without compromising the environment and human health [158]. On the other hand, the main drawbacks of using biopesticides are associated with high susceptibility to climatic conditions, mild pathogens toxicity, and inferior efficacy than conventional pesticides [159].

### 3.7. Textiles

Herb essential oils/extracts have been used in the textile industry, as an alternative to synthetic chemicals and for obtaining materials with antimicrobial, antifungal, and antioxidant activity, which can be used for prophylactic, therapeutic, and protective purposes [103]. Moreover, they represent an eco-friendly alternative for developing innovative and healthcare textiles, since they are safe, widely available, and non-toxic [160]. Herb extracts/essential oils can be applied directly on the textile fibers with or without an immobilization technique or can be immobilized on the textile fiber via encapsulation (Table 4 and Table 5). Salinas et al. [118] tested a polyester textile containing polymeric nanoparticles with glycerin based on oregano essential oil, to promote the fixation of encapsulated oregano essential oil and the increase in the hydrophilicity and comfortability of the fabric sample. The presence of glycerin in the nanocapsules improved the fabric’s comfort. On the other hand, the fabric was not resistant to washing. El-Shafei et al. [102] showed that applying 2% sage extract on the cotton fabric ensures exerting antimicrobial activity against Gram-negative and Gram-positive bacteria and yeasts unicellular fungi, even after ten cycles of washing. Kramar et al. [103] developed a viscose fabric with high antioxidant activity by immersing the fabric sample into a sage ethanolic extract, at a material–liquid ratio of 1:50. Thyme essential oil of different concentrations (5–12% in methanol) was applied to the dry cotton–linen (45–55%), blended fabric, and linen (100%) fabric. The highest antimicrobial (against *Corynebacterium xerosis*, *Bacillus licheniformis*, *Micrococcus luteus*, *Staphylococcus aureus*, *Escherichia coli*, *Klebsiella pneumoniae*, *Pseudomonas aeruginosa*) and antifungal activity was obtained on the cotton–linen blended fabric, when using thyme essential oil of 8% concentration [161]. Despite the high potential of using herb essential oils/extracts for functionalization of the textile materials, further studies are required to improve their durability, productivity, and commercial availability.

## 4. Conclusions

The aromatic herbs from the Lamiaceae family are good sources of bioactive phytochemicals with excellent antioxidant capacity and antimicrobial properties. These biological effects were mainly assigned to the volatile terpenoids and the polyphenolic compounds. The functionality of aromatic herbs is increased when are used in the form of essential oils, extracts, or isolated active compounds. Therefore, all overviewed aromatic herbs have multiple potential applications in various fields like food, feed, cosmetics, biopesticides, and textile industries. In particular, aromatic herbs and their extracts are commonly used for improving the sensory profile of a variety of foods, as well as for extending their shelf life. In particular, the essential oils from rosemary, thyme, and oregano are promising sources of powerful natural antioxidants, to be used as alternatives to synthetic antioxidants for preventing the oxidative deterioration of food products. Regarding antimicrobial activity, thyme essential oil is a promising candidate for eradicating antibiotic-resistant pathogens.

## Figures and Tables

**Table 1:** General details on the cultivating requirements of aromatic herbs [3,4].

Common Name (Botanical Name)		Plant Part Used	Growth Habit and Duration	Climate Zone
Basil (*Ocimum basilicum* L.)	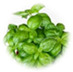	Leaves and terminal shoot	Annual herb. Needs full sun. Grows well in rich and well-drained soil.	Tropical wet and dry, subtropical humid, subtropical dry, or temperate oceanic
Marjoram (*Origanum* *majorana* L.)	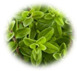	Leaves and floral buds	Perennial herb. Needs full sun. Grows on saline alkaline soil.	Subtropical dry summer, temperate continental, or temperate oceanic
Oregano (*Origanum* *vulgare* L.)	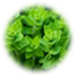	Leaves and flower	Perennial herb. Needs full sun. Grows well in alkaline and well-drained soil.	Steppe or semiarid, subtropical dry summer, or temperate oceanic
Rosemary (*Rosemarinus* *officinalis* L.)	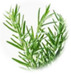	Leaves and terminal shoot	Perennial shrub. Needs full sun. Grows well in sandy and well-limed soil.	Steppe or semiarid, subtropical dry summer, or temperate
Sage (*Salvia officinalis* L.)	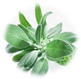	Leaves and terminal shoot	Perennial shrub.	Steppe or semiarid, tropical wet and dry, subtropical dry summer, or temperate
Thyme (*Thymus* *vulgaris* L.)	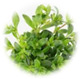	Leaves and terminal shoot	Perennial shrub. Grows well in dry hillsides.	Steppe or semiarid, tropical wet and dry, subtropical dry summer, or temperate
Summer savory (*Satureja* *hortensis* L.)	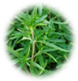	Leaves and terminal shoot	Annual herb. Grows in dry and rocky hillsides.	Steppe or semiarid, subtropical dry summer, temperate

**Table 2:** Chemical composition of the aromatic herbs: details on the main compounds responsible for the nutritive value, flavor, and antioxidant activity.

Aromatic Herb	Details on Nutritive Value (Composition per 100 g)	Main Flavor Compounds	Compounds with Antioxidant Activity	References
Basil	Proteins 14.4 g; Fats 4.0 g; Fiber 17.8 g; Ash 14.3 g Main vitamins: C, B1, B2, A, niacin Main minerals: K, Ca, P, Mg, Fe, Na, Zn	D-Linalool, methyl chavicol, eugenol, cineole	Phenolic acids, flavonoids, especially anthocyanins, as well as eugenol, linalool, estragole, α-cadinol, methyl cinnamate, and α-bergamotene Major *: rosmarinic acid, eugenol, linalool Polyphenol content **: 4318 mg/100 g d.w.	[3,15,16,17,18,19,20,21]
Marjoram	Proteins 12.7 g; Fats 7.0 g; Fiber 18.1 g; Ash 12.1 g Main vitamins: C, A, niacin Main minerals: Ca, K, Mg, P, Fe, Na, Zn	Carvacrol, D-linalool, eugenol, chavicol, methyl chavicol, D-terpineol, caryophyllene limonene, cineol	Phenolic acids, flavonoids (flavanones, flavonols, flavones) Major: ferulic acid, caffeic acid, carnosic acid, carnosol, luteolin-7-O-glucoside and apigenin-7-O-glucoside Polyphenol content: 3846 mg/100 g d.w.	[3,14,15,21,22]
Oregano	Proteins 11.0 g; Fats 10.3 g; Fiber 15.0 g; Ash 7.2 g Main vitamins: vitamins E, B6, B3, B2, B9, B7, A, B1, carotene Main minerals: Ca, K, Mg, P, Fe, Na, Zn, Mn, Cu, Se	Thymol, carvacrol, α-pinene, cineole, linalyl acetate, linalool, dipentene, p-cymene, β-caryophyllene	Derivatives of phenolic acids, flavonoids, tocopherols Major: rosmarinic acid, carvacrol, thymol Polyphenol content: 3117 mg/100 g d.w.	[3,21,23,24,25]
Rosemary	Proteins 4.9 g; Fats 15.2 g; Fiber 17.7 g; Ash 6.5 g Main vitamins: vitamins C, A and B3 Main minerals: Ca, K, Mg, P, Na, Fe, Zn	Cineole, borneol, linalool, eucalyptol, camphor, bornyl acetate, α-pinene, camphene, sabinene, phellandrene, α-terpinene	Phenolic acids, flavonoids (flavanols, flavones), and phenolic terpenes Major: carnosic acid, rosmanol, rosemarinic acid, naringin, carnosol Polyphenol content: 2519 mg/100 g d.w.	[3,21,25,26,27]
Sage	Proteins 10.6 g; Fats 12.7 g; Fiber 18.1 g; Ash 8.0 g Main vitamins: vitamins C and A, niacin Main minerals: Ca, K, Mg, P, Fe, Na, Zn	Thujone, borneol, cineole, bornylesters, α-pinene, salvene, D-camphor phellandrene, ocimene	Phenolic acids and phenolic terpenes Major: rosmarinic acid, carnosic acid, carnosol, rosmanol Polyphenol content: 2920 mg/100 g d.w.	[3,21,28]
Thyme	Proteins 9.1 g; Fats 7.4 g; Fiber 18.6 g; Ash 11.7 g Main vitamins: vitamin A, niacin Main minerals: Ca, K, Mg, P, Fe, Na, Zn	Thymol, carvacrol, linalool, L-borneol, geraniol, amyl alcohol, β-pinene, camphene, p-cymene, caryophyllene, 1,8-cineole	Phenolic acids, flavonoids, as well as terpenes: carvacrol, thymol, p-cymene, caryophyllene, carvone, borneol Major: rosmarinic acid, thymol Polyphenol content: 1815 mg/100 g d.w.	[3,21,29,30]
Summer savory	Proteins 15.0 g; Fats 5.9 g; Fiber 30.7 g; Ash 7.5 g Main vitamins: vitamin A, niacin Main minerals: Ca, K, Mg, P, Fe, Na, Zn	carvacrol, γ-terpinene, p-cymene, pinene, myrcene, α-terpinene, eugenol, β-caryophyllene	Phenolic acids, flavonoids, as well as terpenes: carnosol, carvacrol, thymol Major: rosmarinic acid, thymol and carvacrol Polyphenol content: 4512 mg/100 g d.w.	[21,31,32,33]

* Folin assay (http://phenol-explorer.eu/); ** partially collected from http://phenol-explorer.eu/ (Accessed on 6 February 2025).

**Table 3:** Antimicrobial activity of plant extracts regarding inhibition zone diameters and minimum inhibitory concentration (MIC)/minimum bactericidal concentration (MBC) values against a wide range of pathogenic microorganisms.

Herbs/ Extract or Essential Oils	Data on Antimicrobial Activity				References
	Strains	Inhibition Zone Diameter (mm)	MIC	MBC	
**Basil**					
Essential oil	*S. aureus* ATCC 25923	24.4 ± 1.1	1.5 mg/mL	nd	[36]
		24.0 ± 1.0	0.9 mg/mL		
	*B. subtilis* ATCC 10707	26.1 ± 1.1	0.8 mg/mL		
Dino genotype Essential oil	*S. epidermidis*	11.33 17.00 (Ethanol extract)	nd	nd	[42]
	*E. coli*	17.33			
	*P. aeruginosa*	16.67			
PI 253157 genotype Essential oil	*S. epidermidis*	17.33			
PI 296391 genotype Essential oil	*S. aureus*	17.00			
	*E. coli*	22.00			
‘Genovese’, Blue net treatment Essential oil	*Bacillus cereus*	26.5 ± 3.21	nd	nd	[41]
	*S. aureus*	38.7 ± 1.15			
	*P. vulgaris*	28.0 ± 3.79			
	*C. albicans*	33.7 ± 0.58			
‘Genovese’, Pearl net treatment	*Bacillus subtilis*	26.5 ± 7.18			
	*K. pneumoniae*	34.3 ± 3.44			
Ethanolic extract	*S. aureus*	>20 (+++)	nd	nd	[40]
Essential oil	*C. sakazakii* Lv53	10.1 ± 0.4	0.2% for the eugenol	0.05% (MTC)	[46]
**Marjoram**					
Essential oil	*S. aureus* ATCC 29213	16 ± 0.5	0.125%	nd	[48]
	*S. aureus* MRSA ATCC 43300	14 ± 0.3	0.125%		
	*E. coli* ATCC 25922, *E. coli* AG-100	14 ± 0.4	0.125% 0.250%		
Essential oil	*S. aureus* ATCC 29213	nd	0.156 mg/mL	nd	[49]
	*A. niger* ATCC 16888, *C. albicans* ATCC 18804		0.078 mg/mL		
	*Trichophyton* *mentagrophytes* ATCC 18748, *Aspergillus* *fumigatus* ATCC 96918		0.157 mg/mL		
**Oregano**					
Essential oil	*E. coli* ATCC 25922	20.7	0.49 mg/mL	0.99 mg/mL	[50]
	*S. aureus* ATCC 25923	16.7	1.9 mg/mL	7.9 mg/mL	
	*S. aureus* isolated from a patient	19.7	1.9 mg/mL	3.9 mg/mL	
Commercial essential oil *O. vulgare* var. *hirtum*	clinical sources *L. monocytogenes* L3	nd	1.25 µL/mL	nd	[51]
	L253, L291		0.625 µL/mL		
	L239, L317		0.3125 µL/mL		
	L315		2.5 µL/mL		
	L368		10.0 µL/mL		
SC-CO_2_ extracts of dried, ground	*M. luteus* ATCC 10240, *S. aureus* ATCC 33862, *E. faecalis* ATCC 19433,	nd	0.25 mg/mL	0.25 mg/mL	[52]
	*L. monocytogenes* ATCC 19115		0.25 mg/mL	0.5 mg/mL	
	*E. coli* ATCC 8739, *S. enterica* ser. Enteritidis ATCC 13076		0.5 mg/mL	0.5 mg/mL	
	*P. aeruginosa* ATCC 9027, *C. jejuni* ATCC 33291		0.5 mg/mL	1.0 mg/mL	
**Rosemary**					
SC-CO_2_ extracts from dried leaves	*B. cereus*	nd	0.320 mg/mL	nd	[53]
	*E. faecium*		1.280 mg/mL		
	*E. coli, S. enterica* ser. Enteritidis		2.560 mg/mL		
Commercial essential oil	*A. baumannii* ATCC BAA-747	nd	0.500 mg/mL	nd	[54]
	*C. albicans* ATCC 10231		0.600 mg/mL		
Infusion from dried rosemary leaves	*S. aureus*	nd	≥1.5 mg/mL	nd	[55]
	*P. aeruginosa*	10	≥6.0 mg/mL	nd	
***Salvia* spp. (sage)**					
*S. fructicosa* *(Greece sage)*	*S. aureus* ATCC 29213	18	2.25 mg/mL	4.50 mg/mL	[56]
	*E. coli* ATCC 25922	10	0.563 mg/mL	2.812 mg/mL	
Aerial parts from *S. fruticose* *(Greece sage)*	Methicillin-Sensitive *S. aureus*	17.464 ± 0.253	2.853 mg/mL	nd	[57]
	*S. aureus* MRSA	11.184 ± 0.209			
	*S. aureus* ATCC 29213	9.399 ± 0.148			
*S. officinalis*	*S. aureus*	33.66 ± 5.68	18.75 mg/mL	37.5 mg/mL	[58]
	*P. mirabilis* ATCC 29906	12.33 ± 0.57	0.29 mg/mL	1.17 mg/mL	
	*B. subtilis* ATCC 6633	14 ± 1.73	2.34 mg/mL	4.69 mg/mL	
	*C. albicans* ATCC 10231	25.33 ± 1.15	4.69 mg/mL	9.37 mg/mL	
*S. officinalis* aerial flowering parts	*S. aureus* ATCC 25923	nd	0.125 mg/mL	0.125 mg/mL	[59]
	*P. aeruginosa* ATCC 27853		0.125 mg/mL	4.00 mg/mL	
*S. officinalis* flowering period	*S. pyogenes* ATCC 19615	nd	0.5 mg/mL	0.5 mg/mL	[60]
*S. officinalis* Hydroethanolic extract	*S. faecalis* clinical strain 2	nd	nd	2.1 mg/mL	[61]
	*S. faecalis* clinical strain 2			1.0 mg/mL	
	*S. faecalis* clinical strain 3			8.7 mg/mL	
	*E. faecium* clinical strain 1			2.1 mg/mL	
	*E. faecium* clinical strain 2			4.3 mg/mL	
**Thyme**					
Commercial essential oil	*B. cereus* ESB014	36.1 ± 2.2	0.19%	nd	[62]
	*B. stearothermophilus* ESB016	41.0 ± 4.9	0.39%		
	*C. perfringens* 1.16	22.0 ± 1.0	0.09%		
	*E. faecalis* ATCC 29212	28.9 ± 1.1	0.19%		
	*E. flavescens* DSMZ 7370	30.1 ± 0.4	0.02%		
	*L. monocytogenes* 7946	36.0 ± 1.4	0.05%		
	*S. aureus* MRSA	34.7 ± 3.0	0.09%		
	*A. baumannii* ESB028	32.6 ± 3.7	0.05%		
	*P. mirabilis* ESB027	36.7 ± 1.8	0.05%		
	*S. Tiphymurium* ESB009	29.9 ± 1.8	0.09%		
	*Y. enterocolitica* ESB024	47.8 ± 3.3	0.09%		
	*C. albicans* ESB025	50.7 ± 2.8	0.09%		
Commercial essential oil	*A. butzleri* CCUG 30484	47.5 ± 1.5	>1.024 mg/mL	nd	[63]
	*S. aureus* CCM 4223	41.4 ± 3.7	1.024 mg/mL		
**Summer savory**					
Aqueous extract	*S. aureus* ATCC 6538, *E. faecalis* ATCC 6057, *S. cerevisiae* ATCC 9763	nd	0.250 mg/mL	0.500 mg/mL	[64]
	*K. pneumoniae* ATCC 7881, *E. coli* ATCC 33876	nd	0.250 mg/mL	<0.250 mg/mL	
Hydroalcoholic extract	*L. monocytogenes* ATCC 19114	nd	0.31 mg/mL	0.62 mg/mL	[65]
	*P. aeruginosa* ATCC 27853, *S. typhimurium* ATCC 14028		5.0 mg/mL	10.0 mg/mL	
	*E. coli* ATCC 25922		2.5 mg/mL	5.0 mg/mL	

nd—not determined; MTC—maximum tolerable concentration.

**Table 4:** Applications of herbs products in different fields.

Plant	Extract (E)/Essential Oil (EO)	Type of Product	Properties in the Products	References
**Food**				
Basil	EO	Fermented sausages	The addition of EO reduced the mold growth on the surface of sausages indicating its potential for antifungal protection of fermented sausages against *Penicillium carneum* and *Penicillium polonicum*.	[97]
Marjoram	E	Skimmed yogurt UF-Kariesh cheese	The addition of extract to 1% in yogurt and 2% in cheese contributed to increasing the antioxidant activity while maintaining consumer acceptability in terms of sensorial attributes.	[98]
Oregano	EO	Fish oil	The addition of oregano EO before encapsulation of fish oil by spray-drying increased the oxidative stability during storage.	[99]
Sage	EO	Minced beef meat	Antimicrobial effect against Salmonella sp. Maintained the quality and extended the shelf life of raw or processed meat during refrigeration. Acceptable consumer acceptability of sensorial attributes.	[100]
Thyme, sage	E	Fruit juice	The fruit juices containing 10% aqueous thyme and sage extract showed increased antioxidant activity and superior sensorial attributes.	[101]
Summer savory	EO	Strawberries	Fumigation of strawberry surface with an emulsion containing summer savory essential oil exerted ant-fungal activity against post-harvest pathogenic fungi and extended the shelf life of the strawberries.	[95]
**Textile**				
Sage	E	Cotton fabric	Antibacterial activity against Gram-negative, Gram-positive bacteria and yeasts unicellular fungi.	[102]
Sage	E	Viscose fabric	Direct impregnation of extracts onto the fabric represents an eco-friendly, low-cost disposable medical textile for skin wound treatment.	[103]
**Cosmetics**				
Rosemary	E	Hair lotion	1% herbal hair lotion is an excellent hair growth promoter.	[104]
Tyme, sage, rosemary	E	Shampoo	Enhanced antioxidant properties of samples containing herb extracts while maintaining the technological properties.	[105]
**Feed additive**				
Oregano, sage	EO	Eggs	Increased egg production and reduced the incidence of broken–cracked eggs.	[106]
Rosemary	Freeze-dried	Pork meat	No effect on the sensorial attributes or carcass properties.	[106]
Thyme	Powder	Eggs	Increased the egg weight and yolk color.	[107]
**Biopesticides**				
Rosemary	EO	Twospotted Spider Mite	Rosemary essential oil caused complete mortality of spider mites on greenhouse tomato plants at concentrations that are not phytotoxic to the host plant.	[108]
Thyme	EO	Glyphodes pyloalis W	Thyme EO showed potential to control G. pyloalis larvae in mulberry orchards.	[109]
**Pharmaceuticals**				
Rosemary	E	Anti-inflammatory product	The extract tested in mice using croton oil ear test showed anti-inflammatory activity comparable to indomethacin.	[110]

**Table 5:** Applications using encapsulated herb extracts/essential oils.

Plant	Extract (E)/ Essential Oil (EO)	Wall Material Encapsulation Method	Type of Product	Properties in Products	Reference
**Food**					
Oregano	EO	Sodium alginate Mandarin fiber	Low fat cut cheese	Edible coating containing min. 2% EO enhanced the appearance and the microbial stability of cut cheese resulting in increasing the shelf life.	[117]
Rosemary	E	Alginate Spray-drying	Cottage cheese	Cheese-containing capsules with rosemary extract presented superior antioxidant activity during storage.	[94]
Rosemary	EO	Inulin and whey protein isolate Spray-drying	Minas frescal cheese	The addition of encapsulated essential oil increased the shelf life by controlling the proliferation of mesophilic bacteria.	[114]
Rosemary	E	Soybean protein isolate Freeze-drying	Beef meat fillets	Encapsulation of rosemary extract increased the antimicrobial and antioxidant activity. Nano-encapsulation reduced the microbial and lipid oxidation during storage and extended the shelf life to up to 21 days.	[115]
Thyme	EO	Sodium casein and maltodextrin spray-drying	Hamburger-like meat products	The encapsulated essential oil exerted high antioxidant and antimicrobial activity with promising potential to be used as a preservative in hamburgers.	[116]
**Textile**					
Oregano	EO	Poly-ε-caprolactone nanoparticles nanoprecipitation	Polyester textile	Encapsulation of essential oil improved fabric comfort and promoted antibacterial properties.	[118]
Oregano	EO	Spray-drying	Sports and leisurewear fabrics	Encapsulation delayed the degradation of EO and showed antibacterial protection against E. coli and S. aureus in terms of oil and washings.	[119]
**Biopesticide**					
Basil	EO	β-cyclodextrin co-precipitation	Colorado potato beetle	Spraying the potato plant with encapsulated EO altered the growth, dynamics of development, and proteolytic activity of larvae.	[120]
**Feed additive**					
Basil	EO	Sodium alginate Chitosan	Broiler chicken	Microencapsulation of basil oil showed promising potential for improvement of intestinal integrity and nutrient utilization.	[121]
**Cosmetics**					
Rosemary	EO	Lipid nanoparticles	Gels	Compared with gels containing nonencapsulated essential oil, gels containing 3% rosemary essential oil-loaded lipid nanoparticles applied on skin surfaces for one week, twice a day exerted positive effects on skin hydration and elasticity of volunteers.	[122]

**Table 6:** Applications of herb essential oils on food packaging with edible biodegradable films/coatings.

Plant	Polymer	Food	Properties in Food	Reference
Basil	Starch, cellulose nanofibers	Mandarin orange	Coating prevented weight loss and maintained the quality of the fruits in terms of surface color and pH for up to 12 days of storage at room temperature	[125]
Basil	Chitosan	Cooked ham	Chitosan film was able to reduce the pH increase and the growth of aerobic mesophilic bacteria during storage (10 days, 4 °C)	[126]
Marjoram	Chitosan	Fresh-cut lettuce	Coated lettuce presented antimicrobial activity against total viable counts, yeast and mold counts	[127]
Marjoram	Mung bean protein isolate/pullulan	Minced beef meat	The incorporation of essential oil into films was able to prevent the oxidative degradation of minced beef samples and presented antimicrobial activity against *S. aureus* and *E. coli*	[128]
Oregano	Pectin edible coating	Tomatoes	Coating with essential oil inhibited the growth of Alternaria alternate and promoted the increase in antioxidant activity of the tomatoes	[129]
Oregano	Sodium caseinate, chitosan	Panela cheese	Coating improved the quality and safety of the Panela cheese in terms of microbial growth delay and moisture loss	[130]
Rosemary	Fish myofibrillar protein/chitosan	Fish fillet	The composite film possessed a protective effect on fish muscle by decreasing lipid oxidation during storage	[131]
Rosemary	Whey proteins, cellulose, nanofiber, titanium dioxide nanoparticles	Fresh lamb	The nanocomposite film increased the shelf life of fresh lamb up to 15 days (compared to control) Antimicrobial effect against Gram-positive bacteria	[124]
Rosemary	Chitosan coating	Strawberry	The fungal decay was lower in coated strawberries during storage at 4 °C for 10 days	[132]
Rosemary, oregano	Pectin coating	Broccoli	The use of the two EOs in pectin coating of fresh broccoli showed high antimicrobial activity and antioxidant effect without limiting the sensorial acceptability	[23]
Sage	Chitosan, alginate, gelatin	Carp burgers	Minimum spoilage changes during 20 days of storage Superior sensorial attributes in terms of odor and overall acceptability compared with control	[133]
Thyme	Chitosan coating	Strawberry	Increased shelf life for at least 15 days at 5 °C High stability of physico-chemical and antioxidant properties	[134]
Thyme	Chitosan coating	Avocado	The quality of avocado in terms of color and firmness was protected by coating with chitosan and thyme essential oil Antifungal effect against Colletotrichum gloeosporioides	[135]
Savory	Chitosan coating	Kumquat	Coating prevented weight loss, maintained the vitamin C content during storage for 30 days at 7 °C, with minimum changes in the sensorial properties	[136]

## Data Availability

No new data were created or analyzed in this study. Data sharing is not applicable to this article.
